# Michelangelo and His Prescriptions

**Published:** 1898-03

**Authors:** 


### MICHELANGELO AND HIS PRESCRIPTIONS.

A curious discovery has been made in the Vatican Library, namely, that of a collection of prescriptions and directions for treating various diseases of the eye in the handwriting of Michelangelo. The belief is that the great sculptor, who suffered from some eye trouble, obtained the prescriptions from his medical friends, and then copied them out. The number of the documents in question seems to suggest that Mielangelo made many applications for advice. Possibly he was not satisfied with the results of the treatment, a likely assumption enough in view of the benighted times in which he lived. He died in the year 1546, a period in which but little could have been known of eye diseases and their treatment. But, however this may be, it would be interesting, from the point of view of medieval history, to learn of what the prescriptions consist. Perhaps permission might be obtained to translate them. Their translation would probably reveal many things, and not the least, the fact that Michelangelo was a bold man in following the advice tendered him. —*Med. Press and Circular.*

